# Hydrogen production via aqueous-phase reforming for high-temperature proton exchange membrane fuel cells - a review

**DOI:** 10.12688/openreseurope.13812.2

**Published:** 2021-09-29

**Authors:** Paranjeet Lakhtaria, Paulo Ribeirinha, Werneri Huhtinen, Saara Viik, José Sousa, Adélio Mendes

**Affiliations:** 1 LEPABE - Laboratory for Process Engineering, Environment, Biotechnology and Energy, Faculty of Engineering, University of Porto, Rua Dr. Roberto Frias, Porto, 4200-465, Portugal; 2 VTT Technical Research Center of Finland Ltd, Tietotie 4 C, P.O. Box 1000, FI-02044 VTT, Espoo, Finland; 3 Departamento de Química, Escola de Ciências da Vida e do Ambiente, Universidade de Trás-os-Montes e Alto Douro, Quinta de Prados, Vila Real, 5000-801, Portugal

**Keywords:** Aqueous-phase reforming, methanol, hydrogen, catalyst, fuel cell, PEMFC

## Abstract

Aqueous-phase reforming (APR) can convert methanol and other oxygenated hydrocarbons to hydrogen and carbon dioxide at lower temperatures when compared with the corresponding gas phase process. APR favours the water-gas shift (WGS) reaction and inhibits alkane formation; moreover, it is a simpler and more energy efficient process compared to gas-phase steam reforming. For example, Pt-based catalysts supported on alumina are typically selected for methanol APR, due to their high activity at temperatures of circa 200°C. However, non-noble catalysts such as nickel (Ni) supported on metal-oxides or zeolites are being investigated with promising results in terms of catalytic activity and stability. The development of APR kinetic models and reactor designs is also being addressed to make APR a more attractive process for producing *in situ* hydrogen.

## Abbreviations

APR: aqueous-phase reforming

BET: Brunauer-Emmett-Teller

CHP: combined heat and power

FC: fuel cell

HT-PEMFC: high temperature polymer electrolyte membrane fuel cell

HHV: higher heating value

IWI: incipient wetness impregnation

LT-PEMFC: low temperature polymer electrolyte membrane fuel cell

MeOH: methanol

MEA: membrane electrode assembly

MSR: methanol steam reforming

PR: production rate

WGS: water-gas shift

WHSV: weight hourly space velocity

WI: wetness impregnation

## Nomenclature

Δ *H*° = enthalpy change

*ε*_FP_ = thermal efficiency of fuel processor

*K* = equilibrium constant

*k* = rate constant

*C* = concentration

*R* = reaction rate

## Introduction

Energy decarbonization is critically needed to mitigate climate change and to contribute to economic growth and technological progress. The US Energy Information Administration predicts that between 2018 and 2050, world energy consumption will grow 50% in total and more than 30% in the industrial sector ^1^. According to the United Nations Sustainable Development Goals (goal 7), it is crucial that energy demand is met for the universal access of energy services since 13% of the world’s population still (2019) suffers from a lack of modern energy access ^2^. Fossil fuels such as oil and coal continue to dominate the global energy sector. Consequentially, greenhouse gas emissions, which are pointed to as the main cause of extreme climate change around the world, continues to climb ^3^; the UN recorded 1.3 million climate-related deaths between 1997–2017. This is one of the many reasons to make the transition towards more environmentally friendly and decarbonized energy. Therefore, new sustainable and renewable energy sources are crucial to meet the energy demands created by an increased world population, technological advancements and to mitigate global warming ^4, 5^.

Hydrogen has emerged as a potential energy vector for a clean energy transition ^6^ because it can be produced from a wide range of environmentally friendly sources and can be used for storing energy that is easily convertible into electricity and heat for long periods of time ^4^. Hydrogen can be produced from renewable feedstock such as biomass and synthetic fuels as well as from fossil fuels ^4, 7^. Different technological pathways can be used to produce hydrogen, both for stationary and/or mobile applications. Hydrogen has a high weight energy density of 120 MJ/kg (lower heating value) compared to a traditional fuel such as gasoline (44 MJ/kg), although it has a very low volumetric energy density ^7, 8^. Hydrogen can be used in combustion engines and turbines to produce mechanical energy and in electrochemical processes to produce electricity ^4, 9^. These qualities make hydrogen a promising energy vector. Examples of these are the hydrogen driven trains in Germany, UK and France, and ferries in Norway ^10^. The European Commission has recently ( March 10, 2020) announced the ‘clean hydrogen alliance’ as an initiative to develop hydrogen-based technologies.

Fuel cells are considered to be the next generation of propulsion systems in automotive, portable and stationary applications ^11^. In particular, hydrogen powered proton exchange membrane fuel cells (PEMFCs) are a promising clean power source due to low-emissions, rapid start times and high energy density and efficiency ^12^. Because of the very low volumetric energy density, hydrogen storage and transportation remain a challenge to overcome and a network of hydrogen delivering infrastructure has to be implemented. An inexpensive strategy that fulfils all the requirements for hydrogen storage and transportation has yet to emerge ^4, 7^. Therefore, *in situ* hydrogen production is an exciting approach to overcome the challenges of hydrogen storage and transportation. Currently, the reforming of fossil fuels, such as methane, is the most widely used process to produce hydrogen, representing 71% of total hydrogen produced globally ^5, 13^. Since this source of hydrogen is not renewable, research has shifted toward hydrogen production from renewable sources such as ammonia and alcohols (glycol, methanol and ethanol) ^14^. A large number of studies can be found on catalytic steam reforming of alcohols because of its high hydrogen selectivity and yield ^14– 16^. In addition to steam reforming, aqueous-phase reforming (APR) has emerged recently as a promising competitive approach. Contrary to steam reforming, which employs high temperatures and mild pressures, APR converts aqueous phase organic compounds into hydrogen at lower temperatures. In fact, APR operates at temperatures between 200°C and 250°C; and pressures close to bubble point feedstock, normally from 1.5 MPa to 5.0 MPa ^17^. These operating conditions make APR a potential technology for providing *in situ* hydrogen for automotive and mobile applications, allowing this fuel to be used as a direct hydrogen carrier. This review addresses the early state-of-the-art of APR process for *in situ* hydrogen production, targeting the integration with high temperature (HT)-PEMFCs.

## Methanol APR

In 2002, Dumesic’s research group ^18^ reported APR of an oxygenated hydrocarbon for the first time. Using group VIII metal-based catalysts, this research group successfully demonstrated the APR of sugar and alcohol (e.g. ethylene glycol and methanol) at ca. 226°C in a single reactor. Additionally, these authors proposed a mechanism for hydrogen formation, which became a steppingstone in the APR field ^18^.

APR offers a number of advantages compared with conventional steam or autothermal reforming; APR is more energy efficient, since it eliminates the vaporization of the aqueous/oxygenated hydrocarbon solutions ^19^. Additionally, operating at lower temperatures improves the stability of catalysts, avoiding coke formation, sintering and oxidation ^20^. The low operating temperatures favor the water-gas shift (WGS) reaction, which minimizes the formation of carbon monoxide; and finally, it mitigates undesired decomposition reactions related to carbohydrates ^19^. Contrary to the multi-stage and multi-reactor steam reforming process, APR is an easy single-step and single reactor process ^21, 22^. 

**Figure 1.**
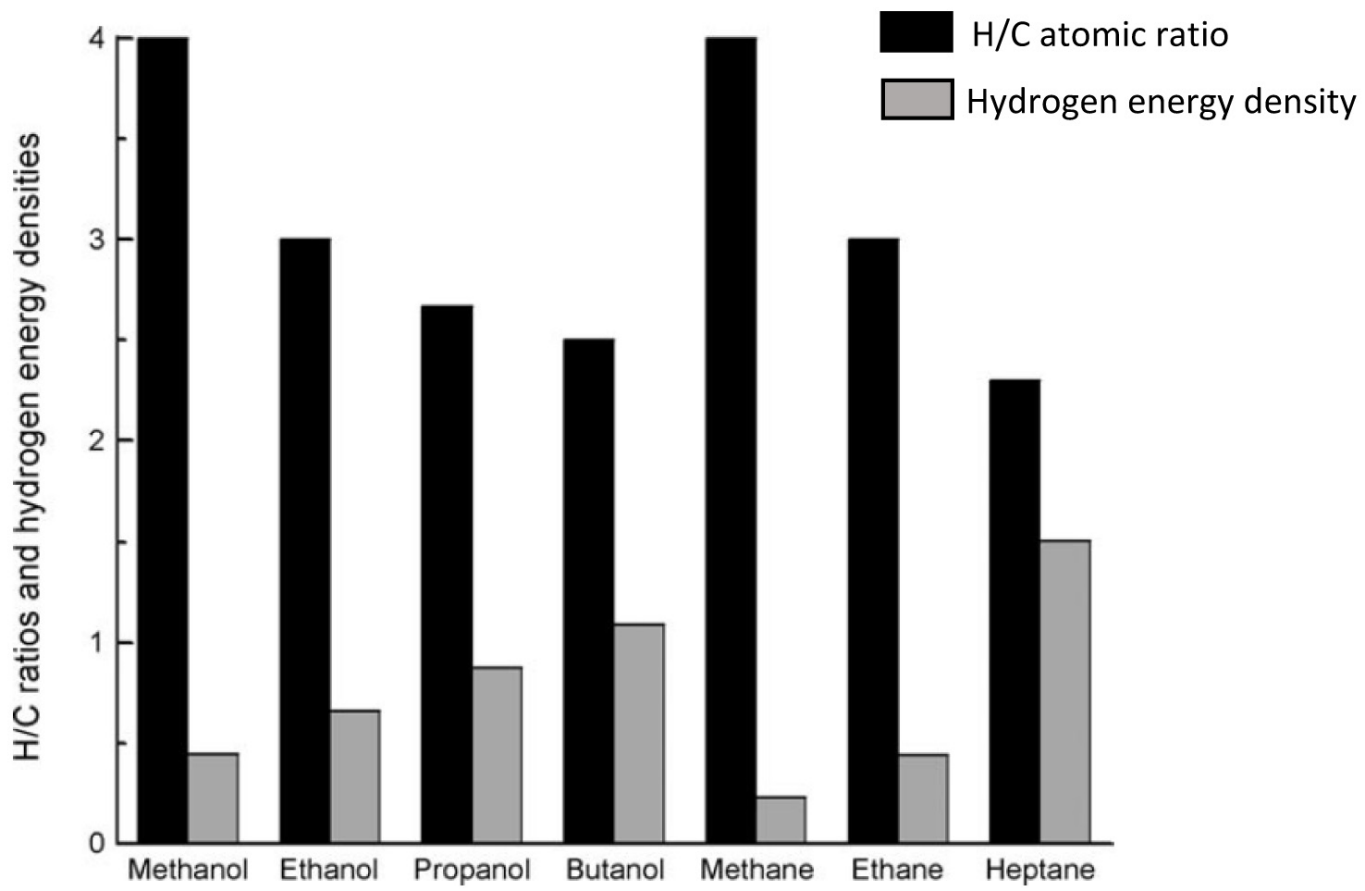
Hydrogen/carbon (H/C) atomic ratio and hydrogen energy densities (10 ^-2^ KJ mol ^-1^ number of H atom ^-1^) of various alcohols and alkanes (reproduced with permission from 25. Copyright 2009. Wiley-VCH Verlag GmbH & Co. KGaA, Weinheim. John Wiley and Sons).

Of all alcohols, methanol is the most convenient hydrogen carrier. With only one carbon, methanol is the simplest alcohol displaying the highest hydrogen to carbon ratio ( Figure 1), meaning that more molecular hydrogen can be extracted from methanol compared to other feedstock ^15, 23^. Methanol displays 12.6 wt. % of hydrogen content, it is liquid at room temperature, it is biodegradable and can be easily produced from biomass and or synthesized ^15^. Methanol reforms – via either steam reforming or APR – at the lowest temperature compared to ethanol or methane, because it has no C-C bonds ^16, 24^.

### Reaction mechanism

The methanol APR mechanism involves cleaving C-H and O-H bonds to produce hydrogen (H _2_) and CO ( Equation 1). The produced CO then reacts with water to form H _2 _and CO _2_ via the WGS reaction ( Equation 2) ^17, 18^.


$${\text{CH}}_{3}\text{OH}⇄2{\text{H}}_{2}+\text{CO}\;\text{ΔH}{}^{\circ}>0\;(1)$$

$$\text{CO}+{\text{H}}_{2}\text{O}⇄{\text{CO}}_{2}+{\text{H}}_{2}\;\text{ΔH}{}^{\circ}<0\;(2)$$

**Figure 2.**
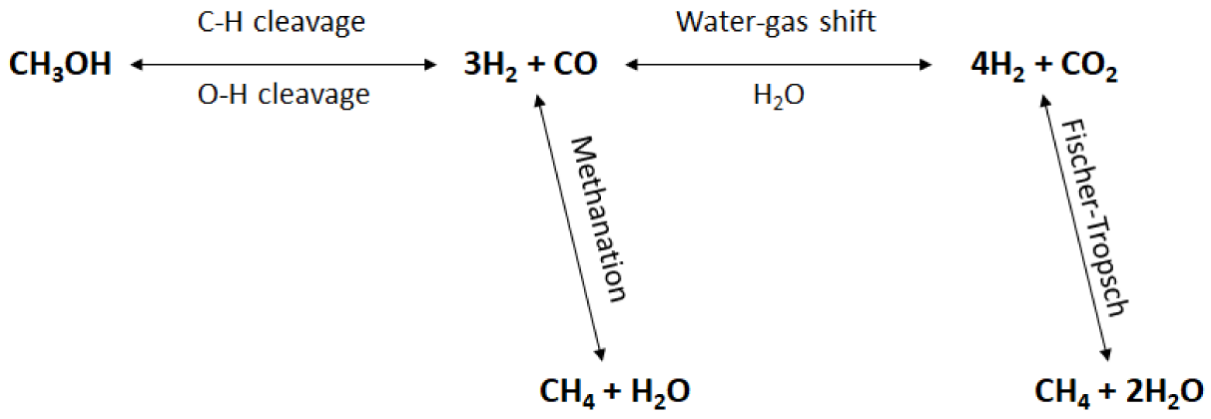
Reaction mechanisms of methanol aqueous-phase reforming (APR).

APR of oxygenated hydrocarbons ( Equation 1– Equation 2) occurs at low temperatures between 200°C and 250°C and are exothermic reactions. However, in both cases ( Equation 1– Equation 2), the reactions thermodynamically favored by higher temperatures. On the other hand, WGS reaction equilibrium ( Equation 2), which converts CO into CO _2_ and H _2_, is favored by lower temperatures and is an endothermic reaction ^17, 18^. The reaction products of APR are broader than just CO _2_ and H _2_. APR conditions thermodynamically favor undesirable side reactions such as methanation and Fischer-Tropsch. The APR operating conditions make carbon oxides thermodynamically unstable, where the cleavage of the C-O bond followed by hydrogenation can form alkanes and water. For methanol APR, methane and water is produced via methanation of CO and Fischer-Tropsch reaction of CO _2_ ( Equation 3– Equation 4) ^17– 19^. Figure 2 shows a schematic representation of methanol APR reaction pathways.


$$\text{CO}+3{\text{H}}_{2}⇄{\text{CH}}_{\text{4}}+{\text{H}}_{2}\text{O}\;\text{ΔH}{}^{\circ}<0\;(3)$$

$${\text{CO}}_{\text{2}}+4{\text{H}}_{2}⇄{\text{CH}}_{\text{4}}+2{\text{H}}_{2}\text{O}\;\text{ΔH}{}^{\circ}<0\;(4)$$

### Catalysts

**Figure 3.**
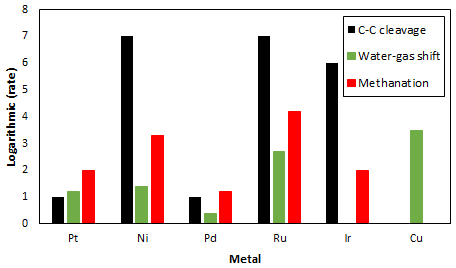
Relative rates for C-C bond cleavage (black) reported in 28, water-gas shift reaction (green) reported in 29 and methanation reaction (red) reported in 35 of various metals.

The focus has been placed on finding suitable catalysts that show high activity for promoting reforming reactions at low temperatures ( Equation 1– Equation 2) and inhibit side reactions ( Equation 3– Equation 4) ^17, 19^. Various catalysts have been used for methanol APR based on noble (Pt, Ru) and non-noble (Ni, Cu) metals, yet the most commonly used catalyst for methanol APR is Pt-based. Dumesic’s group ^18^ made significant advances in APR by using Pt supported on aluminum oxide catalysts. Many authors opted for Pt as it is very active and displays high selectivity towards H _2_^18, 26, 27^. Despite its high catalytic activity for methanol APR, Pt shows lower C-C cleavage activity compared to other metals (Ru, Ni and Ir) ^28^, needed for the APR of heavier alcohols, and reasonable activity for the WGS reaction as shown in Figure 3 ^29^. Additionally, metals such as Cu show the highest activity for WGS but no activity for C-C bond breaking. Different supports, with large surface areas, have also been studied with the objective of increasing the Pt loading in catalysts to increase catalytic activity of Pt-based catalysts ^22, 26, 30^

Nickel-based catalysts have also been investigated for the APR reaction. Although Ni-based catalysts show lower stability and H _2_ selectivity, they favor the WGS shift reaction and are inexpensive compared to Pt. Davda *et al.* ^17^ determined the catalytic activities of various silica supported noble metals (based on the rate of CO _2_ production) for ethylene glycol APR and found that they decrease in the following order: Pt ~ Ni > Ru > Rh ~ Pd > Ir. To improve stability and H _2_ selectivity of Ni-based catalysts, various support materials have been considered ^15, 20, 31– 33^. However, reports on methanol APR via Ni-based catalysts are still limited. Shabaker *et al.* ^34^ and Coronado *et al.* ^31, 32^ made significant advances in this field. The least studied catalysts are of Cu, Ce or Ru. These metal-based catalysts have been used as bi-metallic combinations or their complexes, however. For example, a Ru complex for methanol APR showed high H _2_ yield with turnover frequency of 4700 h ^-1^ and low amounts of unwanted side products (CO and CH _4_) ^36^.

**Figure 4.**
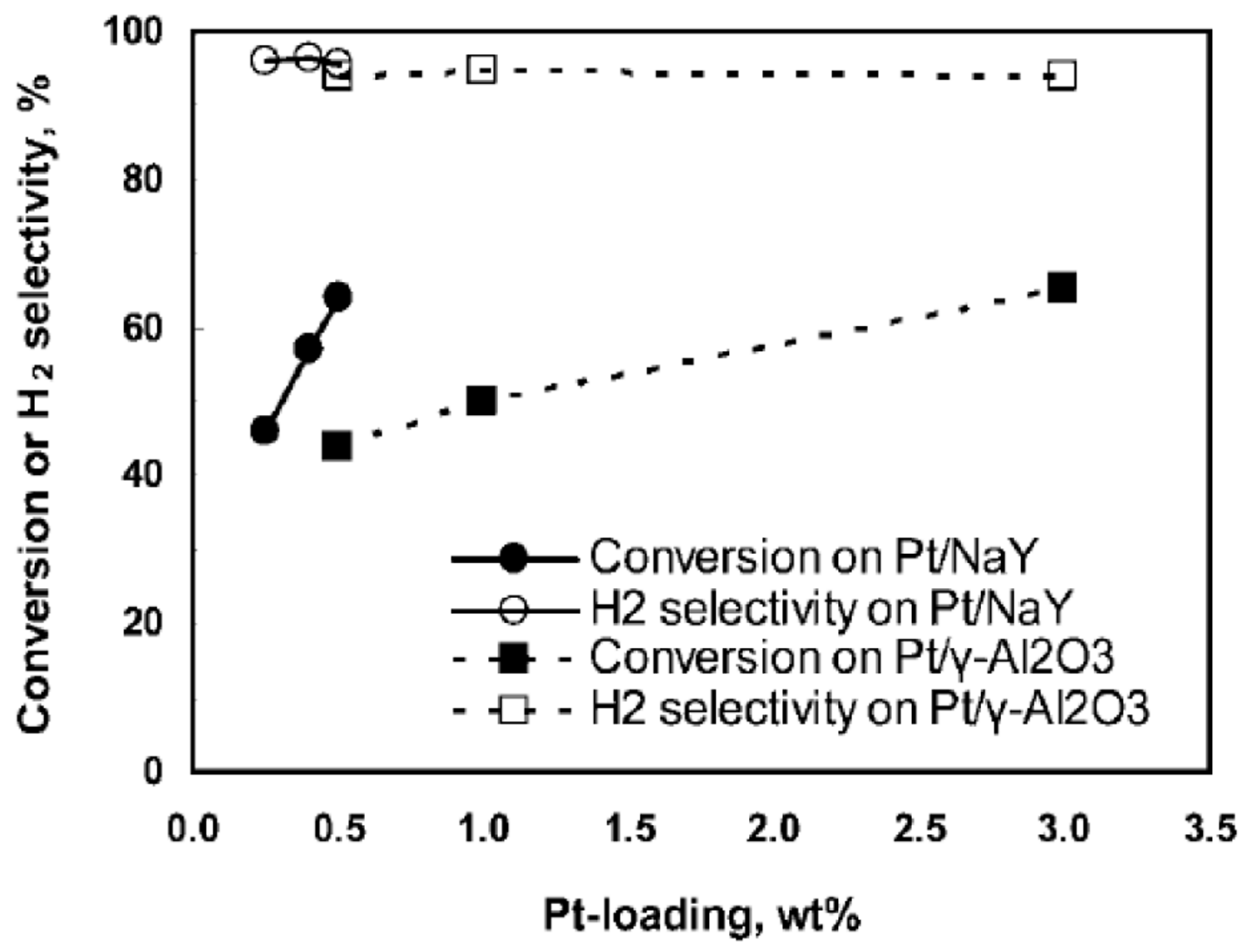
Effect of Pt loading in methanol aqueous-phase reforming. Reprinted with permission from 26. Copyright 2009. American Chemical Society.

***Platinum-based catalysts*.** As previously mentioned, Pt is the favoured metal for APR reactions, showing high activity and selectivity for reforming of oxygenated hydrocarbons ^18^, although low C-C cleavage activity ^17^. To enhance the stability and activity of the Pt-based catalyst, different supports have been investigated such as ZnO, ZrO _2_, TiO _2_ and carbon black ^17^. However, studies on Pt-based catalysts for methanol APR are limited and mostly focused on Pt supported on ɣ-Al _2_O _3_. Shabaker *et al* *.* ^19^ performed a detailed study of methanol APR reaction kinetics using a Pt catalyst supported on alumina (Pt/ɣ-Al _2_O _3_), reporting 100% selectivity for H _2_ concerning alkane formation. ɣ-Al _2_O _3_ showed very high H _2_ yield compared to other supports, followed by α-Al _2_O _3_ and δ-Al _2_O _3_^37^. In contrast, α-Fe _2_O _3_ and CeO _2_ showed low methanol conversion and low H _2_ yield ^37^. Furthermore, Pt/ɣ-Al _2_O _3 _showed improved WGS activity since it had the highest rate of CO _2_ production amongst various Pt-based catalysts. However, under APR reaction conditions, ɣ-Al _2_O _3_ is hydrated to boehmite (AlO(OH)). This reaction reduces the support surface area and promotes the sintering and encapsulation of the Pt nanoparticles. Consequently, it decreases the metal dispersion over the surface of the support, which affects APR performance due to the reduction of the catalyst active sites ^38, 39^. Additionally, it also increases the surface acidity of the catalyst, leading to the formation of undesired side products ^39^. Therefore, it is crucial to find suitable supports for Pt-based catalysts that increase catalytic activity and stability. Following this, Li *et al* *.* ^40^ prepared Pt supported on nickel aluminate (NiAl _2_O _4_) spinel. They observed that the H _2_ yield from Pt/NiAl _2_O _4 _catalyst was four times higher than that of a Pt/Al _2_O _3 _catalyst. The authors attributed the noticeably higher dehydrogenation rate of methanol to the NiAl _2_O _4 _spinel structure and synergy between Pt and NiAl _2_O _4_. The large amount of oxygen vacancies over Pt/NiAl _2_O _4 _stabilize Pt and significantly improved WGS activity. Interestingly, they report negligible activity of NiAl _2_O _4 _in APR tests, which indicates clearly a synergistic effect between Pt and NiAl _2_O _4_. The performance and stability of this catalyst for APR is also affected by the oxidation state of Pt on NiAl _2_O _4_, which should be reduced to metallic Pt from PtO _x_. Zeolites have also been tested as a potential support for methanol APR catalysts ^26^. Pt supported on NaY zeolite shows a slight increase in methanol conversion and H _2_ selectivity compared with a Pt/Al _2_O _3 _catalyst ^26^. This fact was attributed to the synergistic effect between Pt and NaY, where the high electron density of the microporous zeolite surface stabilizes Pt and enhances the hydrogen retention capacity in Pt/NaY catalysts ^26^. The highest H _2_ production rate reported was achieved using Pt supported on various structures of molybdenum carbide (α-MoC, β-MoC), as can be seen in Table 1 ^27^. Lin *et al* *.* ^27^ using density functional theory (DFT) calculations showed that the enhanced APR activity is due to high interaction between Pt and MoC caused by highly atomically dispersed Pt atoms on the MoC surface. Increasing Pt loading usually leads to higher H _2 _yield and production rate. They observed a sharp increase in H _2 _production rate when Pt loading increased from 2 wt. % to 2.2 wt. % ^27^. Higher Pt load leads to the increased number of active sites for the reaction. Figure 4 shows the methanol conversion as a function of the Pt load. 

**Table 1.** Pt-based catalyst for hydrogen production in methanol aqueous-phase reforming (IWI – incipient wetness impregnation, WI – wetness impregnation, WHSV – weight hourly space velocity).

Support	Pt loading (wt. %)	Preparation method	Temperature (°C)	Pressure (MPa)	Methanol concentration (wt. %)	WHSV (h ^-1^)	Methanol conversion (%)	H _2_ production rate (µmol/min/ g _catalyst_)	Ref.
**ɣ- Al _2_O _3_**	3	IWI	225	2.5	1	0.008	94	11.6	18
**ɣ- Al _2_O _3_**	3	IWI	267	2.5	1	0.008	94	11.6	18
**Al _2_O _3_**	2	WI	190	6				294	27
**ɣ- Al _2_O _3_**		IWI					13.1	197	37
**ɣ-Al _2_O _3_**	8	IWI	230	3.2	5	2		41.2	32
**ɣ- Al _2_O _3_**	3	IWI	224	2.65	1	0.008	78.9	9.5	26
**ɣ- Al _2_O _3_**	3	IWI	264	2.65	1	0.008	95.5	11.8	26
**ɣ-Al _2_O _3_**	1	IWI	210	2.9	10	2.94	26.5	107.3	40
**δ- Al _2_O _3_**		IWI					13.8	164	37
**α- Al _2_O _3_**		IWI					10.5	145	37
**CeO _2_**		IWI					3.8	41	37
**α-Fe _2_O _3_**		IWI					4.5	24	37
**α-MoC**	2.2	WI	190	6				7776	27
**α-MoC**	2	WI	170	6				3000	27
**α-MoC**	2	WI	150	6				924	27
**α-MoC**	0.2	WI	190	6				4572	27
**β-MoC**	2.1	WI	190	6				300	27
**MoC-2**	2	WI	190	6				4092	27
**MoC-3**	2.3	WI	190	6				636	27
**NaY**	0.5		224	2.65	1	0.08	81.0	34.8	26
**NaY**	0.5		264	2.65	1	0.08	98.8	44.4	26
**NiAl _2_O _4_**	1	IWI	210	2.9	10	2.94	99.9	439.2	40
**TiO _2_**	2	WI	190	6				252	27

***Ni-based catalysts*.** Ni-based catalysts have been investigated for the APR process, since they show appreciable H _2_ production and are less expensive than Pt-based catalysts. Table 2 summarizes the Ni-based catalysts reported in the literature for methanol APR. Ni-based catalysts are more susceptible to oxidation in hydrothermal APR conditions ^41^. Additionally, sintering, agglomeration and coke formation leads to the deactivation of Ni-based catalysts ^39, 42^. To overcome these issues, Ni has been supported on various materials to increase H _2_ production along with stability. Raney nickel offers a notably high surface area, thermal and structural stability; adding Sn does not influence the reaction rate of Raney nickel catalysts, but increases the selectivity for H _2 _by weakening CO and H adsorption, which lowers the methanation reaction rate ^34^. The rate of H _2_ production, selectivity and stability of Raney NiSn is comparable to Pt/Al _2_O _3 _catalysts. Ni has been supported on metal oxides, such as Al _2_O _3_ and SiO _2_, but also on CeO _2_ and ZrO _2 _( Table 2). It should be emphasised that both CeO _2_ and ZrO _2_ have catalytic activity themselves, making them attractive for APR. For instance, ZrO _2_ enhances WGS activity ^43^. The use of mixed oxides (bimetallic supports) such as CeO _2_-ZrO _2_ and La _2_O _3_-ZrO _2_ improved methanol conversion, hydrogen production and catalyst stability ^44^. The addition of CeO _2_ increases oxygen storage capacity, catalyst mobility and promotes redox properties. Ni supported on [CeO _2_] _0.25_-[ZrO _2_] _0.75_, mass fractions showed superior performance compared to Ni supported on [CeO _2_] _0.17_-[ZrO _2_] _0.83_ or compared to a Ni/ZrO _2_ catalyst ^44^. However, compared to the performance of Ni supported on CeO _2_-ZrO _2_, Ni/CeO _2_ based catalysts showed worse results; poor metal dispersion due to a lesser surface area was pointed to as the main reason ^44, 45^. Ni has also been tested as a catalyst without using any support material. Shabaker *et al.* ^34^ used a Sn modified Ni catalyst for reforming various hydrocarbons and concluded that adding Sn was required to supress the alkane formation reaction. Nevertheless, Ni without any support showed better H _2_ selectivity since it avoids the often-observed dehydrogenation of the catalyst support.

**Table 2.** Ni-based catalysts for hydrogen production in methanol aqueous-phase reforming (IWI – incipient wetness impregnation, WI – wetness impregnation, WHSV – weight hourly space velocity).

Support	Ni loading (wt. %)	Preparation method	Temperature (°C)	Pressure (MPa)	Methanol conversion (%)	WHSV (h ^-1^)	H _2_ yield (%)	H _2_ production rate (µmol/min/ g _catalyst_)	Ref.
**ZrO _2_**	10	IWI	230	3.2		2	2	20.3	32
**ZrO _2_**	7	IWI	230	3.2	48	4	40		45
**ZrO _2_**	10	IWI	230	3.2	26.4	3.6		1021	44
**[CeO _2_] _0.17_-[ZrO _2_] _0.83_**	10	IWI	230	3.2	50.9	3.6		2144	44
**[CeO _2_] _0.25_-[ZrO _2_] _0.75_**	10	IWI	230	3.2	58.8	3.6		2529	44
**[CeO _2_] _0.17_-[La _2_O _3_] _0.05_-** **[ZrO _2_] _0.78_**	10	IWI	230	3.2	45.7	3.6		2134	44
**[La _2_O _3_] _0.10_-[ZrO _2_] _0.90_**	10	IWI	230	3.2	46.6	3.6		2147	44
**Calcia-stabilized** **ZrO _2_**	5	IWI	230	3.2	75	4	64		45
**Yttria-stabilized ZrO _2_**	5.6	IWI	230	3.2	46	4	36		45
**Calcia-stabilized** **ZrO _2_**	6.9	IWI	230	3.2	63	4	46		45
**CeO2**	10	IWI	230	3.2	16.9	3.6		453	44
**α-Al _2_O _3_**	11.8	IWI	230	3.2		2	0.8	8.6	32
**SiO _2_**	10	IWI	230	3.2		2	1	10.1	32
**ɣ-Al _2_O _3_**	11.4	IWI	230	3.2		2	6	60.6	32
	R-NiSn 14		225	2.5	18.6		1.43		34

To increase basicity of the APR reaction and consequently the selectivity for H _2_, calcia-stabilized ZrO _2_ (CSZ) and yttria-stabilized ZrO _2_ (YSZ) have been tested as supports ^45^. The results showed that adding calcia to ZrO _2_ improves the APR performance with higher methanol conversion and H _2_ yield, while yttria-stabilized ZrO _2_ had no impact on performance. However, increasing the loading of calcia for CSZ did not necessarily increase H _2_ production. Lanthanum (La) has been employed with Ce and Zr to increase WGS activity and catalyst stability. Adding La _2_O _3_ to a CeO _2 _support increases oxygen vacancies, provides better metal-support interactions and leads to higher H _2_ production due to better Ni dispersion ^44^ .

***Effect of dopants*.** Adding Cu to Ni-based catalysts supported on Al _2_O _3 _and SiO _2_ significantly increases WGS activity and makes methane formation less favorable, as depicted in Table 3 ^32^. This behavior is less evident in other types of support. Coronado *et al.* ^31^, using CeO _2_-ZrO _2 _supports, reported higher H _2_ selectivity for alcohol dehydrogenation for Ni-Cu/[CeO _2_] _0.25_-[ZrO _2_] _0.75_, in comparison to Ni/[CeO _2_] _0.25_-[ZrO _2_] _0.75_, despite the lower methanol conversion and H _2_ yield. The authors justified the lower performance with the lower loading of nickel in Ni-Cu/[CeO _2_] _0.25_-[ZrO _2_] _0.75_. On the other hand, adding Ce to Ni catalysts supported on Al _2_O _3 _and SiO _2_ showed slight improvements in methanol conversion ^32^. The opposite effect was observed for CeO _2_-ZrO _2 _supports ^31^, due to an increase in the nickel particles’ size. Similar results were obtained by Goma *et al.* ^45^, using a Ni-based catalyst doped with CeO _2_ over different supports (ZrO _2_, calcia-stabilized ZrO _2_ and yttria-stabilized ZrO _2_). The results showed that the CeO _2 _doped catalysts have lower performances than those without CeO _2_. The reduction properties and basic nature of Ce reduces the CO selectivity. Moreover, the CeO _2_ layer additionally hindered the basic sites of calcia and yttria, lowering the methanol conversation and H _2_ production.

**Table 3.** Ce and Cu doped Ni-based catalysts for hydrogen production in methanol aqueous-phase reforming at 230°C and 3.2 MPa (5 wt. % methanol concentration) (IWI – incipient wetness impregnation, WI – wetness impregnation, WHSV – weight hourly space velocity).

Metal	Support	Metal loading (wt. %)	Preparation method	Methanol conversion (%)	WHSV (h ^-1^)	H _2_ yield (%)	H _2_ prodution rate (µmol/min/ g _catalyst_)	Ref.
**Ni-Cu**	[CeO _2_] _0.25_-[ZrO _2_] _0.75_	Ni 10 Cu 5	IWI	38	3.6	101	2100	31
**Ni-Cu**	SiO _2_	Ni 10 Cu 5	IWI		2	1.0	10.9	32
**Ni-Cu**	Al _2_O _3_	Ni 13 Cu 1.3	IWI		2	6.2	63.5	32
**Ni-Ce**	[CeO _2_] _0.25_-[ZrO _2_] _0.75_	Ni 10 Ce 5	IWI	12	3.6	18	620	31
**Ni-Ce**	Al _2_O _3_	Ni 13 Ce 1.3	IWI	19	3.6	28	950	31
**Ni-Ce**	SiO _2_	Ni 10 Cu 5	IWI		2	3.4	34.19	32
**Ni-Ce**	Al _2_O _3_	Ni 13 Cu 1.3	IWI		2	8.3	84.7	32
**Ni-Ce**	ZrO _2_	Ni 5.9 Ce 12.6	IWI	40	4	34		45
**Ni-Ce**	Calcia-stabilized ZrO _2_	Ni 5.9 Ce 13	IWI	68	4	57		45
**Ni-Ce**	Calcia-stabilized ZrO _2_	Ni 5.5 Ce 13.5	IWI	44	4	33		45
**Ni-Ce**	Yttria-stabilized ZrO _2_	Ni 5.5 Ce 12.7	IWI	54	4	40		45

***Noble metal-based catalysts*.** Besides Pt-based catalysts, other noble metals such as Ru, Ir, Rh and Pd have been employed in APR of alcohols ^17, 20^, but only Ru and Ir were studied for methanol APR. The dehydrogenation of methanol at low temperatures between 89°C and 95°C, using Ru-based pincer complexes in alkaline solution, showed that decreasing the base concentration (NaOH) negatively affects the catalytic performance ^36^. The presence of formate as an intermediate to produce H _2_ and CO _2 _was identified by Raman spectroscopy. The use of the Ru complex allows high H _2_ yield with a turnover frequency of 4700 h ^-1^ and low amounts of CO and CH _4_ with long-term stability ^36, 46^. Ir-based catalysts supported on SiO _2_, TiO _2_ and ZrO _2_ have also been used in methanol APR. Adding Re to Ir/SiO _2_ and Ir/ZrO _2_ increased the H _2_ formation rate by four times and five times, respectively ^47^, by forming nanocomposite clusters that decreased the H _2_ adsorption sites on Ir. Meanwhile, adding Mo to Ir/TiO _2_ created a thin monolayer of MoO _2_ on TiO _2_ that is responsible for improving the water reactivity. This hydration process over the monolayer leads to an increased H _2_ production rate.

**Figure 5.**
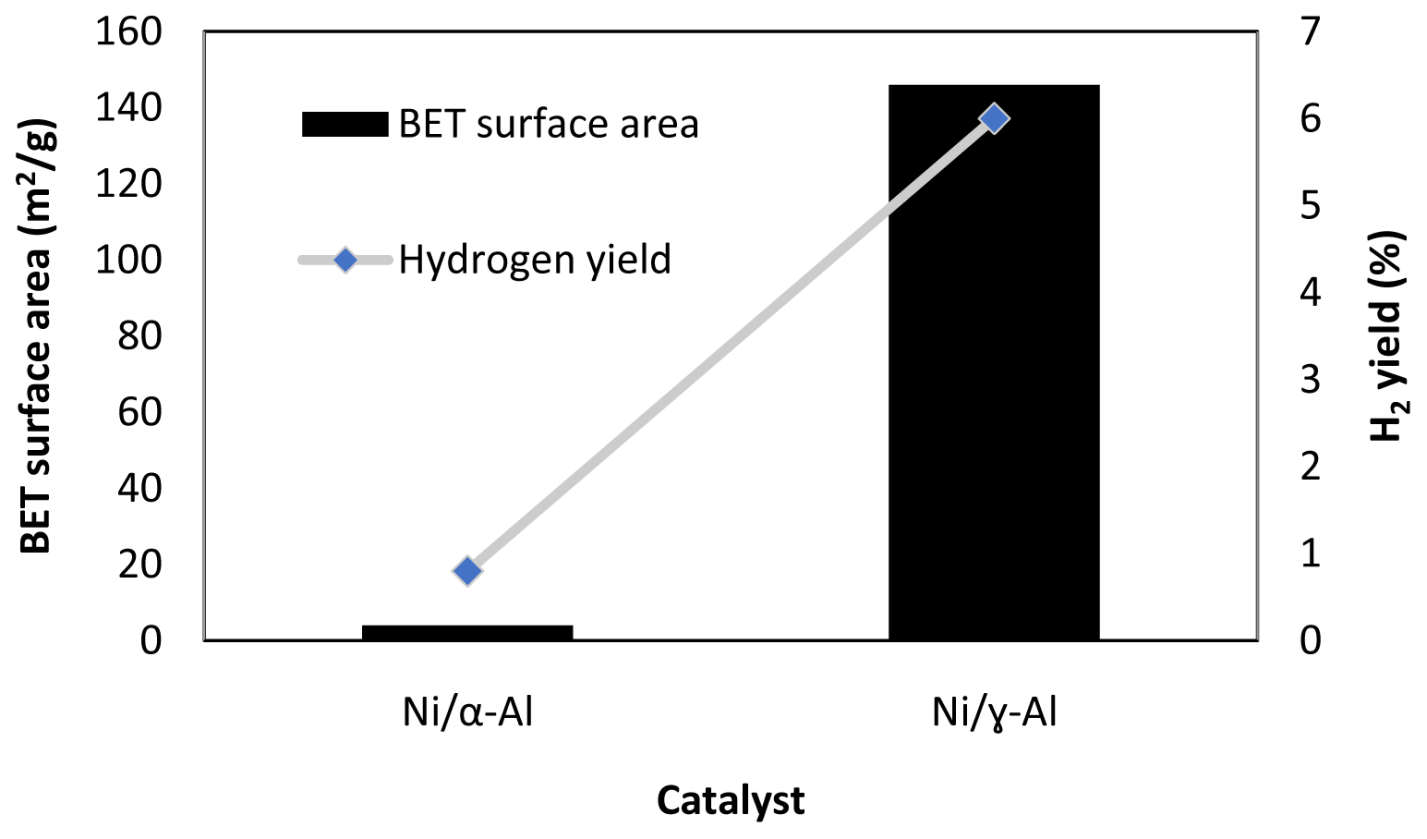
Catalyst Brunauer–Emmett–Teller (BET) surface area and hydrogen yield in methanol aqueous-phase reforming, 230°C, 3.2 MPa, WHSV 2 h ^-1^, Ni loading 11.8 wt. % for Ni/α-Al _2_O _3_ catalyst, Ni loading 13 wt. % for Ni/ɣ-Al _2_O _3_ (figure created using data obtained from 32).

***Supports and preparation methods*.** The performance of the catalyst relies on the active surface area of the support, the dispersion of the metal over the support surface and the size of the active metal nanoparticles. Therefore, it is necessary to optimize the preparation method in order to achieve catalysts with highly dispersed metal over the support surface. The most commonly used methods for catalyst preparation are derivations from co-precipitation and wet-impregnation. It is important to opt for an appropriate aforementioned preparation method (depending on metal precursors and supports) in order to achieve optimal catalytic activity. Incipient wetness impregnation (IWI) is a commonly used method for preparation of catalysts for methanol APR. However, the use of metal nitrates often offers poor dispersion of the catalyst. Studies on Fe and Ni precursors attributed the reason for low dispersion to redistribution during the drying process. Adding viscosity-increasing agents or chelating citrate salts to aqueous nitrate solutions can avoid redistribution problems ^48, 49^ Figure 5 shows one example of the effect of surface area in methanol APR. As seen in this figure, the use of an alumina support with a larger surface area (α-Al _2_O _3_) corresponds to higher H _2_ yield. The reason behind this could be that the larger surface area of the support allows for a better dispersion of Ni than for the support with lower surface area (ɣ-Al _2_O _3_).

***Alkane formation*.** In APR, methanation and Fischer-Tropsch reactions are responsible for alkane formation. For APR of methanol, only CH _4_ is formed as side product alkane. The formation of undesired CH _4_ can be suppressed to some extent by using suitable metal and/or support materials. Alkane formation heavily depends on the acidity of the catalyst material. Additionally, using catalysts of basic nature not only suppresses alkane formation, it is also favorable for WGS activity ^50^. Huber *et al.* ^51^ reported that alkane selectivity increased as acidic sites were added to non-acidic Pt/Al catalyst.

As previously mentioned, different metals have their own methanation activity rate (Ni shows a higher tendency to form alkanes than Pt). In order to decrease alkane selectivity and favor higher H _2_ formation, non-acidic dopants or supports must be used with a Ni-based catalyst during dehydrogenation reactions. Not only the metal of the catalyst, but also the support plays an equally vital part in APR reactions. Acidic supports like SiO _2_-Al _2_O _3_ tend to favor higher alkane formation compared to basic or neutral supports such as Al _2_O _3_^17^. Ni-based catalysts have been doped with Cu in order to increase H _2_ production and to suppress CH _4_ formation; however, adding Cu had no noticeable effect on increasing H _2_ production ^32^. Use of a Ce doped Ni-based catalyst also limits CH _4_ formation, which could be the result of a synergetic effect between Ni and Ce ^31^. Calcia and yttria have been added to ZrO _2_ in Ni-based catalysts to increase the surface basicity. This resulted in promoted WGS activity that decreased the selectivity of alkane formation ^45^. Table 4 summarizes secondary substances producing rate on catalysts used in MeOH APR. It is clear from this Table that Ni-based catalysts display higher alkane rate production than the Pt-based catalysts. This is in accordance with Figure 3 that indicates that Ni displays a higher methanation rate than Pt.

**Table 4.** Alkane (CH _4_ and C _2_H _6_) producing rate on various methanol aqueous-phase reforming catalysts. (PR – production rate, NO – not observed).

Metal	Support	Metal loading (wt. %)	Temperature (°C)	Pressure (MPa)	WHSV (h ^-1^)	CH _4_ yield (%)	CH _4_ (mol. %)	CH _4_ PR (µmol/ min/g _catalyst_)	CH _4_ selectivity (%)	C _2_H _6_ yield (%)	C _2_H _6_ PR (µmol/min/ g _catalyst_)	Ref.
**Pt**	ɣ- Al _2_O _3_	3	225	2.5	0.008		0.4				0.0	18
**Pt**	ɣ- Al _2_O _3_	3	267	2.5	0.008		0.6				0.0	18
**Pt**	Al _2_O _3_	2	190	6								27
**Pt**	ɣ- Al _2_O _3_							0.97				37
**Pt**	ɣ-Al _2_O _3_	8	230	3.2	2	0.1		0.9		NO	NO	32
**Pt**	ɣ- Al _2_O _3_	3	224	2.65	0.008		0.5					26
**Pt**	ɣ- Al _2_O _3_	3	264	2.65	0.008		0.6					26
**Pt**	ɣ-Al _2_O _3_	1	210	2.9	2.94							40
**Pt**	δ- Al _2_O _3_							1.01				37
**Pt**	α- Al _2_O _3_							1.10				37
**Pt**	CeO _2_							0.20				37
**Pt**	α-Fe _2_O _3_							0.02				37
**Pt**	α-MoC	2.2	190	6								27
**Pt**	α-MoC	2	170	6								27
**Pt**	α-MoC	2	150	6								27
**Pt**	α-MoC	0.2	190	6								27
**Pt**	β-MoC	2.1	190	6								27
**Pt**	MoC-2	2	190	6								27
**Pt**	MoC-3	2.3	190	6								27
**Pt**	NaY	0.5	224	2.65	0.08		0.6					26
**Pt**	NaY	0.5	264	2.65	0.08		0.8					26
**Pt**	NiAl2O4	1	210	2.9	2.94							40
**Pt**	TiO _2_	2	190	6								27
**Ni**	ZrO _2_	10	230	3.2	2	1.3		13.4		0.1	1.5	32
**Ni**	ZrO _2_	7	230	3.2	4				2.2			43
**Ni**	ZrO _2_	10	230	3.2	3.6				1.5			42
**Ni**	[CeO _2_] _0.17_-[ZrO _2_] _0.83_	10	230	3.2	3.6				2.2			42
**Ni**	[CeO _2_] _0.25_-[ZrO _2_] _0.75_	10	230	3.2	3.6				3.3			42
**Ni**	[CeO _2_] _0.17_- [La _2_O _3_]0.05- [ZrO _2_] _0.78_	10	230	3.2	3.6				2.0			42
**Ni**	[La _2_O _3_] _0.10_- [ZrO _2_] _0.90_	10	230	3.2	3.6				2.6			42
**Ni**	Calcia- stabilized ZrO _2_	5	230	3.2	4				2.1			43
**Ni**	Yttria- stabilized ZrO _2_	5.6	230	3.2	4				1.5			43
**Ni**	Calcia- stabilized ZrO _2_	6.9	230	3.2	4				2.1			43
**Ni**	CeO2	10	230	3.2	3.6				0.0			42
**Ni**	α-Al _2_O _3_	11.8	230	3.2	2	0.3		3.4				32
**Ni**	SiO _2_	10	230	3.2	2	0.2		2.5				32
**Ni**	ɣ-Al _2_O _3_	11.4	230	3.2	2	3.3		33.4		0.2	2.1	32
**Ni-Cu**	[CeO _2_] _0.25_- [ZrO _2_] _0.75_	Ni 10 Cu 5	230	3.2	3.6							31
**Ni-Cu**	SiO _2_	Ni 10 Cu 5	230	3.2	2	0.0		0.0		NO	NO	32
**Ni-Cu**	Al _2_O _3_	Ni 13 Cu 1.3	230	3.2	2	0.7		7.3		0.0	0.1	32
**Ni-Ce**	[CeO _2_] _0.25_- [ZrO _2_] _0.75_	Ni 10 Ce 5	230	3.2	3.6							31
**Ni-Ce**	Al _2_O _3_	Ni 13 Ce 1.3	230	3.2	3.6							31
**Ni-Ce**	SiO _2_	Ni 10 Cu 5	230	3.2	2	0.7		7.4		0.0	0.2	32
**Ni-Ce**	Al _2_O _3_	Ni 13 Cu 1.3	230	3.2	2	3.2		32.1		0.2	2.0	32
**Ni-Ce**	ZrO _2_	Ni 5.9 Ce 12.6	230	3.2	4				0.9			43
**Ni-Ce**	Calcia- stabilized ZrO _2_	Ni 5.9 Ce 13	230	3.2	4				2.2			43
**Ni-Ce**	Calcia- stabilized ZrO _2_	Ni 5.5 Ce 13.5	230	3.2	4				2.5			43
**Ni-Ce**	Yttria- tabilized ZrO _2_	Ni 5.5 Ce 12.7	230	3.2	4				0.9			43

### Effect of experimental conditions

Methanol APR can be carried out in the range 200°C – 250°C within the pressure range of 1.5 MPa – 3.5 MPa. Therefore, it becomes necessary to observe the effect of system pressure and temperature for optimized H _2_ production. Pan *et al*. studied the effect of pressure on H _2_ production along with the production rates of CO _2_ and CH _4_ in APR of 5 wt. % ethylene glycol aqueous solution for Ni _20_SnAl _7_ hydrotalcite derived catalyst. It was reported that H _2_ production decreased significantly with increased pressure (from 2.6 MPa to 3.0 MPa) whereas the production rate of CH _4_ was unaffected. The decrease in CO _2_ production was also observed with increasing operating pressure. This effect was ascribed to blockage of available catalytic sites and results in dehydration and hydrogenation of intermediate products at the expense of produced H _2_. This results in the decrease of H _2_ production ^52^. 

Additionally, partial H _2_ pressure becomes a limiting factor to H _2_ production. Methanol APR forms H _2_, CO, CO _2_ and sometimes CH _4_ in gaseous form, which is in the form of gas bubbles within the aqueous solution. The total pressure inside the bubble is calculated to be the same as the total system pressure considering vapor-liquid equilibrium ^19^. The high H _2_ pressure blocks catalytic sites and lowers the surface concentration of CO, which reduces the activity of the WGS reaction. Additionally, changes in total system pressure influence the gaseous bubble density and size distribution. Such changes affect the performance of the catalyst by modifying their wetting behavior ^53^. In the same manner as pressure, system temperature also influences H _2_ production. For example, at high temperature water can corrode the catalyst and active metal and/or support can be washed away into the effluent ^19^. However, thermodynamic analysis of methanol APR show higher H _2_ selectivity at higher temperatures (>187°C). On the contrary, CH _4_ formation decreases with temperature increase (and increases with pressure increase). Since CH _4_ formation is an exothermic process, the methanation reaction is thermodynamic unfavored by higher temperatures ^54^.

### Reactor types

**Figure 6.**
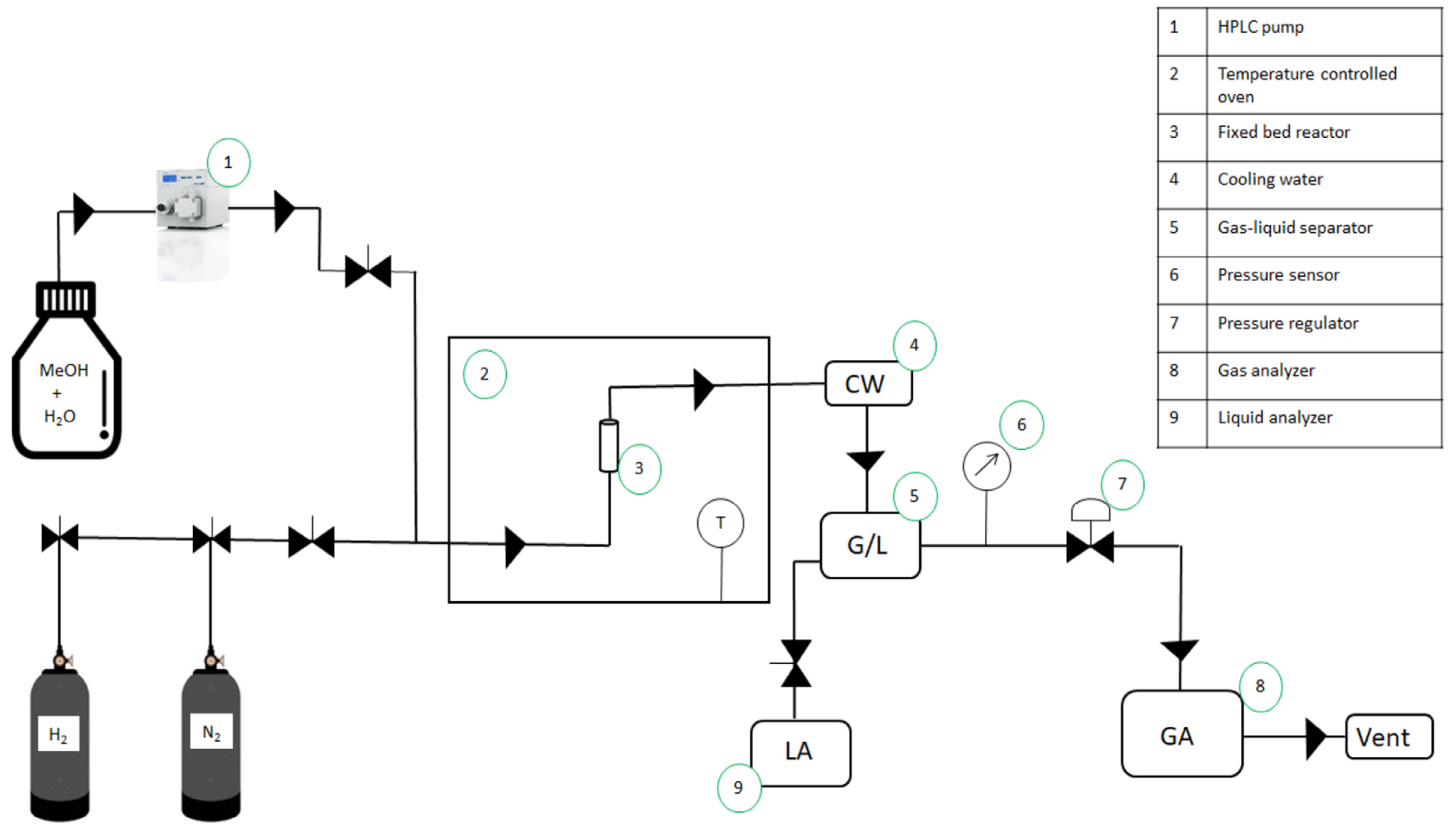
Schematic representation of methanol aqueous-phase reforming experimental set-up.

The catalyst and reactor design must be accurately selected for obtaining the best APR performance. APR is a three phase reaction which reaction kinetic is often controlled by the diffusion of reactants and products in the liquid phase. Namely hydrogen, which has a very low solubility in methanol and in water, diffuses slowly from the reaction center where it is formed to the gas phase, outside the catalyst; the reaction is then normally controlled by diffusion ^34^. Additionally, the evolved hydrogen may take part in the methanation and/or Fischer-Tropsch side reactions that further decreases the H2 production rate ^34^. Therefore, catalyst and reactor should be designed to facilitate the fast hydrogen removal from to maximize the hydrogen production rate. Most studies consider the use of fixed packed reactors for MeOH APR, but this design is certainly not the best suitable for this reaction. In a typical lab-scale APR experimental set-up, an aqueous MeOH solution is fed upwards in a packed bed reactor using an HPLC pump. Before starting the reaction, whenever needed hydrogen is used as a reducing medium for catalyst reduction. Normally nitrogen, but also argon, are then used as sweep gas as to strip away the produced hydrogen. A condenser is used to separate the gas products from the unreacted methanol and water. The products are analyzed with an online gas chromatograph and unreacted reactants can be analyzed with a liquid chromatograph. A pressure regulator is used to maintain the operating pressure of APR such as methanal and water are in liquid phase. Figure 6 shows a schematic representation of a typical MeOH APR reaction set-up using a fix bed reactor.

Microchannel and membrane reactors have also been considered for the APR reaction. These reactors allow higher mass transfer rates of reactants and products and then higher selectivities and hydrogen production rates. D’Angelo *et al*. published very interesting studies on the use of carbon-coated ceramic membrane reactors ^55^ and coated microchannel reactors ^56^ for APR of sorbitol and ethylene glycol, respectively. The carbon-coated ceramic membrane showed 2.5 times higher hydrogen yield when compared with the fixed bed reactor tested under similar experimental conditions. Since some metals selective possess H _2_ permeability, due to this fact, membrane reactor can be facilitated as both reaction and purification system in reforming reactors ^57, 58^. The membrane reactor facilitates the effective removal of hydrogen from the reaction medium due to the differential pressure between retentate and permeate. This product removal from reaction system can increase H2 yield. Additionally, the authors pointed that the hydrophobic nature of membrane hinders its interaction with water, therefore increasing stability of membrane. The coated microchannel reactor displayed a similar performance when compared with the membrane reactor ^55^. On another fascinating study, D’Angelo *et al*. ^56^ coated Pt/AlO(OH) catalyst particles on the inner wall of a microchannel (inner diameter of 320 µm) to perform the APR of ethylene glycol. This single microchannel reactor showed increased hydrogen selectivity and ethylene glycol conversion compared to the fixed bed reactor performance used under identical conditions. The authors attributed this greater performance to the hydrodynamic behavior under Taylor flow regime, which facilitates the removal of hydrogen from catalyst surface; such reactors should also be assessed for MeOH APR.

### APR kinetics and modeling

Kinetic modelling is a way to shed light on the reaction pathways leading to rate of formation of the products of various catalysts at given temperatures. Ribeirinha *et al.* ^59^ studied empirical and mechanistic models for the high performance catalyst CuO/ZnO/Ga _2_O _3 _for methanol steam reforming (MSR). Simulating a packed bed reactor, they found that these models accurately fit to the experimental results, after appropriate parameter estimation. Lotrič *et al.* ^60^ developed a power-law empirical model for the same catalyst CuO/ZnO/Ga _2_O _3_ and reported good fitting with experimental data. There are several kinetic models reported in the literature for MSR for various catalysts, reaction temperatures and types of reactor ^61– 67^ In contrast, APR kinetic models are scarce as the technology is still in its development phase. Coronado *et al.* ^68^ presented a kinetic model for C _1_-C _4_ alcohols derived from Fischer-Tropsch synthesis over Ni supported on ceria-zirconia using power law and phenomenological models. The authors evaluated three different reaction rate equations. The first equation was a Langmuir–Hinshelwood reaction rate expression based on the work by Murzin *et al.* ^69^ for xylitol APR ( Equation 5), and the two other equations ( Equation 6– Equation 7) were simple power law models.


$$\;R_{A}=k\frac{C_{alcohol}}{\left(1+K_{alcohol}C_{alcohol}\right)}\;(5)$$

$$\;R_{B}=kC_{alcohol}^{m}\;(6)$$

$$\;R_{C}=kC_{alcohol}\;(7)$$

The authors considered only alcohols as being able to adsorb on the active sites of the catalyst. The adsorption equilibrium constants of alcohols ( *K*), the rate constants ( *k*) and the reaction orders ( *m*) were considered as lumped parameters to avoid system over-parametrization. Water concentration was considered constant and the WGS reaction was assumed to be dependent on the concentration of CO and H _2_O. The Arrhenius and Van’t Hoff equations were used to calculate the temperature dependency of the reaction rate constants and adsorption equilibrium constants, respectively ^68^. However, the availability of APR kinetics modelling is still limited and in its preliminary stage. Further study and development in the field of APR kinetic models are required in order to better understand APR reaction kinetics that can help optimize and upscale APR.

## Feasibility of reforming for fuel cell applications

Low-temperature PEMFCs (LT-PEMFCs) such as Nafion based polymer electrolyte membrane fuel cells are state-of-the-art fuel cells that operate below 100°C. Despite the excellent proton conductivity, the poor CO tolerance at the anode, slow oxygen reduction reaction kinetics and complex water management system hinders cost reduction and performance improvement of LT-PEMFCs. Some studies reveal that LT-PEMFCs suffer significant performance loss in the presence of > 10-20 ppm of CO in fuel feed ^70^. Operating at temperatures above 100°C has the benefits of enhanced catalytic activities and simplified water management. HT-PEMFCs are also more tolerant to fuel impurities and have low dependency on heat removal systems ^71, 72^. HT-PEMFCs also have a better CO tolerance compared to LT-PEMFCs. A HT-PEMFC operating at 130°C can tolerate up to 1000 ppm of CO compared to LT-PEMFCs operating temperature 80°C, which can tolerate only up to 10 ppm of CO ^70, 73^. Such attributes of HT-PEMFCs provide the potential of *in situ* hydrogen production using fuel processors based on reforming or oxidation processes. One of the most important reasons for *in situ* H _2_ production is the non-availability of a viable H _2_ storage material ^74– 76^. In this sense, *in situ* H _2_ production has been extensively researched in recent years as a source of H _2 _feedstock for fuel cells. The integration of fuel cells with a steam reformer is thermally advantageous since fuel cells are exothermic, while reformers are endothermic. Several studies investigating the thermal integration of low temperature steam reforming with HT-PEMFCs can be found in the literature ^23, 77– 81^. However, the operating temperature remains a hurdle to overcome in the integration of steam reformers and HT-PEMFCs. HT-PEMFCs have the operating temperature range of 120°C – 200°C ^82^, whereas the steam reforming of alcohols occurs at temperatures between 150°C and 500°C ^83– 85^. The operating temperature for state-of-the-art HT-PEMFCs cannot exceed 200°C, as it would lead to problems such as degradation and loss of proton conductivity. Additionally, the temperature difference between HT-PEMFCs and steam reformers is more of a challenge for rapid startup ^86, 87^.

### Fuel processors for fuel cells applications

**Figure 7.**
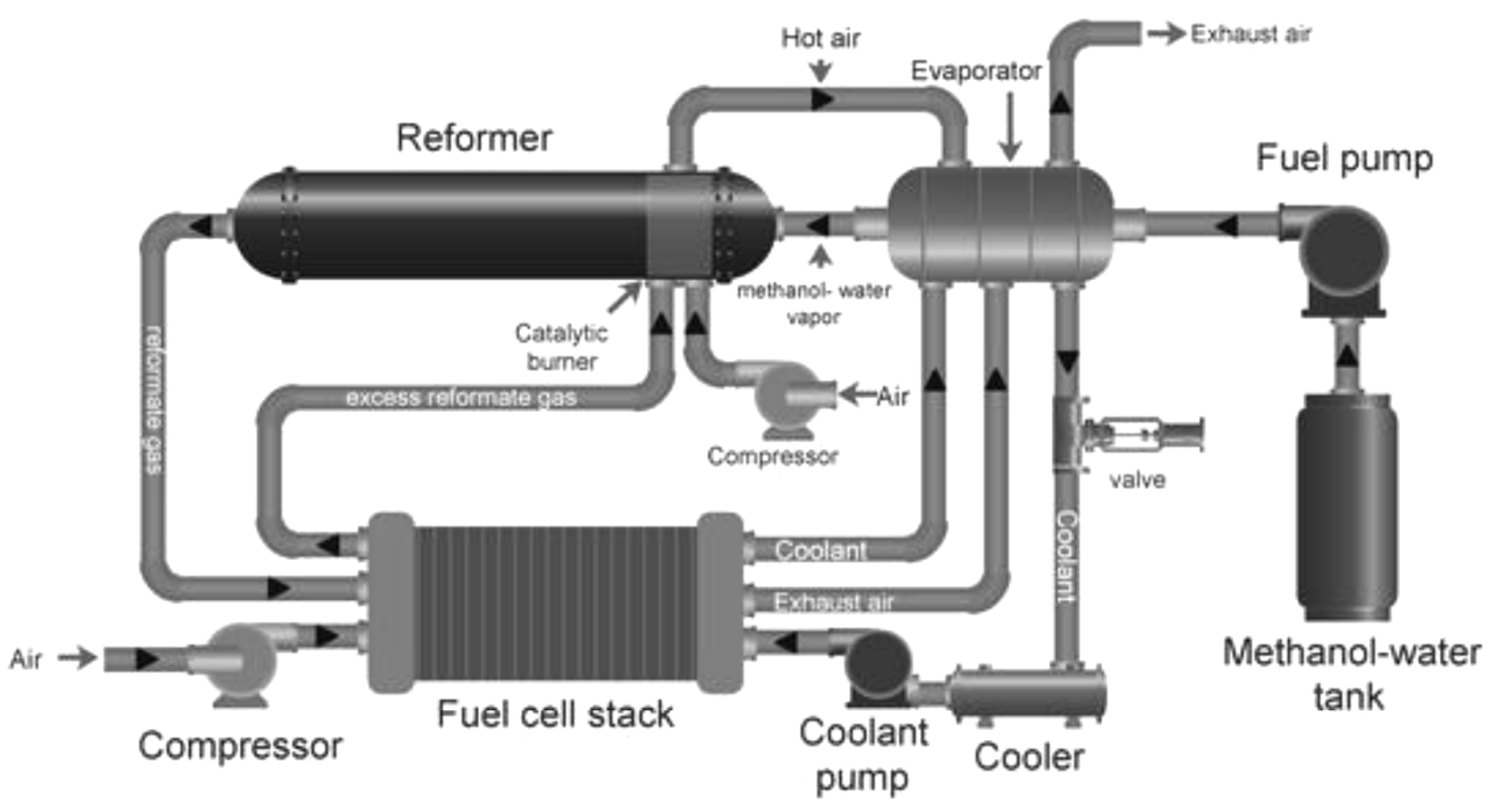
Schematic diagram of an integrated fuel (methanol) processor with fuel cell. Reproduced from 93 (open access, Creative Commons Attribution (CC BY 4.0) license. Licensee MDPI, Basel, Switzerland.).

Fuel processors are complex devices that convert hydrocarbons into H _2_. A typical fuel processor consists of a steam generator, preheater, catalytic fuel reformer, WGS reactor, exhausters, condenser and catalytic after burner ( Figure 7). Catalytic fuel reforming is undoubtedly the most crucial part of the cogeneration system as it produces H _2_ for the fuel cell. A vaporizer or heater is another important component of the fuel processor that is required to heat fuel and water to meet the catalytic reaction conditions of the hydrocarbon. Many fuel processers are equipped with CO removal systems, such as WGS reactors ^88, 89^. When using LT-PEMFCs, it becomes critical to feed in pure H _2_ to avoid the contamination of the fuel cell anode catalyst; therefore, more complex purification systems are required, such as pressure-swing adsorption (PSA) units. Since methanol reforming involves production of CO _2_, it is critical to install a CO _2_ capture system at the outlet to avoid adding to CO _2_ emissions ^90– 92^. 

Fuel processors make the overall HT/LT-PEMFCs a complicated system, as it is vital to maintain the temperatures of various subsystems. This in turn can make the thermal integration of the system a challenging task (especially for relatively small fuel cell systems) since the temperatures for different subsystems can vary across wide ranges. The thermal efficiency of a fuel processor ( *ε*_FP_) ( Equation 8) is calculated using the higher heating value (HHV) of H _2_ produced via reforming divided by the HHV of the input fuel (e.g. methanol). The total thermal efficiency of the fuel processor is brought down by the energy required to run the fuel processor subsystem (e.g. pumps, condenser, exhaust fans, etc.). Brett *et al*. ^89^ reported that if one counts the (electrical) efficiency of the whole system, in reality it is one third to one fifth lower than the efficiency counted for only the fuel cell stack.


$$\;\varepsilon_{FP}(\%)=\frac{\Delta H_{(HHV),H_{2}}}{\Delta H_{(HHV),fuel}}\;(8)$$

### APR integration with HT-PEMFC

Internal reforming of methanol or integration of a methanol reformer with a fuel cell is distinctly advantageous compared to fuel processor systems due to the complex thermal management, water management and eventual loss in overall efficiency due to many subsystem components. To overcome this issue, researchers have studied lowering the temperature of MSR for integration with HT-PEMFC ^24, 94– 96^. Lotrič *et al*. ^80^ followed a different approach, by considering a higher HT-PEMFC stack operating temperature of 255°C. The authors investigated the integration of MSR with fuel cells and reported that HT-PEMFC shows higher system efficiency than LT-PEMFC in an integrated system. However, operating fuel cell stacks at 255°C requires novel electrolytes capable of proton conduction at such a temperature in absence of water, using expensive gaskets to withstand high temperatures, and development of a new catalyst support, as widely used carbon supports corrode faster at high temperatures ^88^.

These issues can be avoided, to some extent, via methanol APR. Integrating methanol APR with HT-PEMFCs can be seen as a potential technology to increase efficiency and to overcome the complexity of MSR integration into HT-PEMFCs. As discussed earlier, methanol APR, using catalysts such as Pt/MoC, can be performed at temperatures below 200°C with considerably high H _2_ production ^27^. Additionally, APR is an exothermic process, which makes its integration with fuel cell a simpler and more efficient method of H _2_ and electricity production ^80^. Using methanol APR with HT-PEMFCs is advantageous since the undesired byproducts of reforming are easy to control, except for CH _4_ and CO, where the concentration is below the limits of HT-PEMFC tolerance. Integrating APR with HT-PEMFCs can also make the whole system more compact since methanol APR is an easy single-step, single reactor process. The methanol APR reaction conditions are far better suited for integration with HT-PEMFCs than MSR. APR integrated HT-PEMFCs have potential for higher CHP fuel cell system efficiency and performance than MSR integrated HT-PEMFCs.

***Challenges with integration*.** Although the integration of APR and HT-PEMFCs has several advantages, there are also distinct barriers. Challenges are related to the low maturity of the APR technology, elementary APR catalyst research, and the feasible conditions of the integrated system.

To improve the overall efficiency of the HT-PEMFC system and to make the integration of APR and HT-PEMFCs more attractive, the waste heat recovered from the FC stack should be utilized as the heat source of the APR unit. In thermal integration, the waste heat of the FC stack is utilized with a heat transfer fluid recycled in the overall system. Thermal integration would enhance the benefits of APR and compensate for the lower methanol conversions and hydrogen production rates compared to the high values attained with MSR technology. Due to thermal losses in the heat recovery process, the operation temperature of the APR unit is below the operation temperature of the FC stack. Moreover, even though the operation temperature of the HT-PEMFC may vary in the range of 120–200°C, the degradation of the stack is faster at higher operation temperatures ^97^. Therefore, in the thermal integration of APR and HT-PEMFC technology, the selection of operation temperature is based on the optimization of APR catalyst activity and stack lifetime.

Since the reforming temperature should remain below 200°C, a major challenge is the lack of data related to APR systems at decreased temperatures. As presented in Table 1 and Table 2, the methanol conversion and hydrogen production rate drop when the reforming temperature is decreased. A high conversion rate is essential for the system to reach high system efficiency. Furthermore, the anode of the HT-PEMFC tolerates only limited amounts of vaporized feed solution; even a 3% volume fraction of methanol-water vapor would degrade the FC performance ^98^. To avoid this, either a more efficient catalyst or a cold trap for vapor collection is necessary for the integrated system.

Additionally, a challenge with the APR catalysts is the lack of long-term activity experiments. Li *et al.* ^27^ reached excellent hydrogen production rates with a Pt/α-MoC catalyst, but their experiments lasted less than two hours, which provides no information on long-term catalyst activity. When the APR unit is integrated with the HT-PEMFC stack, the stability of the APR catalyst should correspond with the durability of the HT-PEM membrane electrode assemblies (MEAs), which have recently reached a lifetime of 9,000 hours without any problems ^99^. *In situ* re-reduction of deactivated catalyst demands more complexity from the FC system and therefore it should be avoided if possible.

Even though high-pressure hydrogen storage is not required when hydrogen is produced *in situ* with APR, the pressure conditions of APR are relatively high. To perform APR, the reforming pressure should be above the bubble point pressure of the feed mixture. If the methanol molar fraction in the feed solution is 30%, the bubble point pressure of the solution is over 9 bar at 150°C and over 29 bar at 200°C ^100, 101^. Since the HT-PEMFC is operated at around atmospheric pressure, the reforming pressure should be maintained with a back-pressure regulator.

In three-phase reforming systems, such as APR, mass transfer limitations cause challenges. Typically, they are solved with nitrogen co-feeding, which enhances the removal of hydrogen molecules from the porous catalyst surfaces. Nevertheless, when the APR is integrated with the HT-PEMFC, nitrogen co-feeding would dilute the feed gas, which is unfavorable. The separation of nitrogen and hydrogen is possible with novel membrane separation techniques ^102, 103^. However, such a separation system would increase the complexity and capital expenditure (CAPEX) of the FC system, and thus is not considered a reasonable solution. Instead, usage of a micro-channel reactor or novel catalyst coating on a structured catalyst have been suggested to improve the mass transfer properties of the APR system ^20^.

## Conclusion

In recent years, research on methanol APR for hydrogen production has been increasing, with a focus on developing new catalysts. Pt-based catalysts are the best performing catalysts and the highest hydrogen production was reported using MoC and NiAl _2_O _4_ as supports. Unconventional supports such as Zeolite and MoC performed better than conventional supports such as alumina and silica. Non-noble metal-based catalysts, such as Ni-based catalysts, show promising results and are the focus of most methanol APR catalytic developments. Ni has been supported on various metal oxides such as CeO _2_ and ZrO _2_, which increased overall hydrogen production by promoting WGS reaction. The addition of Cu to produce a bi-metallic Ni-based catalyst leads to higher WGS activity and suppressed methanation, whereas adding Ce only leads to increased hydrogen yield. Ru, Re and Ir are other less frequently used metals that showed promising results and need to be further explored.

Methanol APR has the potential to be considered as a prominent hydrogen production technology. However, methanol APR using non-noble metal catalysts need further development to reach Pt-based catalyst activity, where the supports play a major role. Furthermore, kinetic models need to be developed for better understanding of APR reaction mechanisms, as well as being important tools for scaling, designing and optimizing processes.

## Data availability

No data are associated with this article.
