# Supplementary material for: Hydrogen production via aqueous-phase reforming for high-temperature proton exchange membrane fuel cells - a review
Source: Open Res Eur. 2022 Mar 23;1:81. Originally published 2021 Jul 20. [Version 3] doi: 10.12688/openreseurope.13812.3 (PMC10445907; doi:10.12688/openreseurope.13812.3)
Supplement: Peer review history

# Peer review: Reviewer response for version 3

**DOI:** 10.21956/openreseurope.15827.r28914

**Published:** 2022-04-21

**Authors:** Ying Li

**Affiliations:** 1 Institute of Industrial Catalysis, College of chemical engineering, Zhejiang University of Technology, Hangzhou, China

The authors have revised the manuscript according to my review. I approve it to be indexed.

Is the review written in accessible language?

Yes

Are all factual statements correct and adequately supported by citations?

Yes

Are the conclusions drawn appropriate in the context of the current research literature?

Yes

Is the topic of the review discussed comprehensively in the context of the current literature?

Yes

Reviewer Expertise:

Catalysis

I confirm that I have read this submission and believe that I have an appropriate level of expertise to confirm that it is of an acceptable scientific standard.

# Peer review: Reviewer response for version 2

**DOI:** 10.21956/openreseurope.15285.r28087

**Published:** 2022-03-09

**Authors:** Ying Li

**Affiliations:** 1 Institute of Industrial Catalysis, College of chemical engineering, Zhejiang University of Technology, Hangzhou, China

This manuscript reviews the aqueous-phase reforming, mainly introducing the Pt, Ni-based catalysts and other noble metal catalysts. Although it is with clear classification and a comprehensive introduction, there are minor issues that need to be revised.

1. In the manuscript, except for the review of APR, the feasibility of reforming for fuel cell applications is also demonstrated in Page 14-15. I suggest this part can be also presented in the Abstract.
2. In the part of “Supports and preparation methods”, it is mainly described the effect of preparation methods. Therefore, the “support” in the subtitle is suggested to be removed.
3. In the part of “Alkane formation”, the effects on the APT mainly derive from the metal species. I think it is reasonable to change the subtitle into the “Effects of active metal” or other that about the metals. It seems to show better consistency of the context by this modification.
4. In the last part of the conclusion, more outlook and challenges of the APR can be added.

Is the review written in accessible language?

Yes

Are all factual statements correct and adequately supported by citations?

Yes

Are the conclusions drawn appropriate in the context of the current research literature?

Yes

Is the topic of the review discussed comprehensively in the context of the current literature?

Yes

Reviewer Expertise:

catalysis

I confirm that I have read this submission and believe that I have an appropriate level of expertise to confirm that it is of an acceptable scientific standard, however I have significant reservations, as outlined above.

# Response

**Published:** 2022-03-21

**Authors:** Paranjeet Lakhtaria

**Affiliations:** 24337, Portugal

The authors acknowledge these comments. The discussion section has been improved as recommended

1. As suggested, the abstract has been updated by including the information regarding the feasibility of fuel cell application and APR integration with HT-PEMFC.
2. The heading of subsection “Supports and preparation methods” has been changed to “catalyst synthesis methods” for the better understanding of the provided information.
3. The authors acknowledge this suggestions. The subtitle “alkane formation” has been changed with “effect of active metal on alkane formation”.
4. Conclusion has been updated with more outlook and challenges faced with APR technology as recommended.

# Peer review: Reviewer response for version 2

**DOI:** 10.21956/openreseurope.15285.r27716

**Published:** 2021-09-30

**Authors:** Adolfo Iulianelli

**Affiliations:** 1 Institute on Membrane Technology of the Italian National Research Council (CNR-ITM), University of Calabria, Rende, Italy

The Authors have taken into consideration all my revision requests and the manuscript has been improved accordingly. Therefore, from my side I suggest to accept it for indexing.

 Nevertheless, I report below some minor corrections to be done:

- The acronyms PSA, CSZ, YSZ, DFT are missing in the list of Abbreviations. Please, add them.
- In various Tables, the Authors should revise various terms such as: CH ^4^, H ^2^, H ^2^ production ^catalyst^, WHSV ^-1^) (h, etc.  For instance, the link below contains an useful picture of Table 1 where I highlighted something needing revisions.

Table 1 Revisions.

Is the review written in accessible language?

Yes

Are all factual statements correct and adequately supported by citations?

No

Are the conclusions drawn appropriate in the context of the current research literature?

Yes

Is the topic of the review discussed comprehensively in the context of the current literature?

Yes

Reviewer Expertise:

NA

I confirm that I have read this submission and believe that I have an appropriate level of expertise to confirm that it is of an acceptable scientific standard.

# Peer review: Reviewer response for version 1

**DOI:** 10.21956/openreseurope.14891.r27272

**Published:** 2021-08-09

**Authors:** Adolfo Iulianelli

**Affiliations:** 1 Institute on Membrane Technology of the Italian National Research Council (CNR-ITM), University of Calabria, Rende, Italy

The review manuscript deals with an interesting overview about the advantages due to the combination of the aqueous phase reforming of methanol process with high temperature PEM fuel cells. It describes carefully the role of the various catalysts, the kinetics and the benefits of the aqueous phase reforming of methanol for hydrogen generation compared to the gas phase process. The main comment is related to the absence of discussion about the potential of the membrane reactor technology utilization to carry out the aqueous phase reforming reactions. It is suggested to add a paragraph on the membrane reactor technology and the possible benefits due to its utilization in the aqueous phase reforming of methanol process, based on the examples present in literature for other reaction processes such as “ *Neira D’Angelo, M. F., Ordomsky, Van der Schaaf,J., Schouten, J. C., & Nijhuis, T. A. (2014). Carbon-coated ceramic membrane reactor for the production of hydrogen via aqueous phase reforming of sorbitol. ChemSusChem, 7(7), 2007-15*”.

 In the following, a few minor comments are reported further:

 1. A schematic about the aqueous phase reforming reactor should be added and discussed. Furthermore, a diagram of the APR reactor integrated to the HT-PEMFC should be provided like to Fig. 6.

 2. The CO tolerance for both LT and HT-PEMFCs should be reported numerically in the paragraph “Feasibility of reforming for fuel cell applications”.

 3. Regarding to the main performance of the APR reactors present in literature, information about the by-products compositions should be added (a dedicated table is suggested).

 4. Regarding to the sentence “ *However, the operating temperature remains a hurdle to overcome in the integration of steam reformers and HT-PEMFCs. HT-PEMFCs have the operating temperature range of 120°C – 180°C, whereas the steam reforming of alcohols occurs at temperatures between 220°C and 500°C ^76,77^*” reported in the paragraph “Feasibility of reforming for fuel cell applications”, neither ref. 76 nor 77 refer to the range of temperatures for carrying out the steam reforming of alcohols as reported in the aforementioned sentence.

 The following references are suggested to be used instead of ref. 77:

- Amiri *et al.* - Chemical Engineering & Processing: Process Intensification, 157 (2020) 108148 ^1^.
- Palma *et al*. – Catalysts, 10 (2020) 665 ^2^.

5. The quality of Figure 4 should be improved.

 6. A list of symbols and acronyms should be added.

Is the review written in accessible language?

Yes

Are all factual statements correct and adequately supported by citations?

No

Are the conclusions drawn appropriate in the context of the current research literature?

Yes

Is the topic of the review discussed comprehensively in the context of the current literature?

Yes

Reviewer Expertise:

Hydrogen generation by reforming reactions; catalytic membrane reactors for hydrogen production; fuel cells; membrane gas separation.

I confirm that I have read this submission and believe that I have an appropriate level of expertise to confirm that it is of an acceptable scientific standard, however I have significant reservations, as outlined above.

## References

[1] : Membrane reactors for sustainable hydrogen production through steam reforming of hydrocarbons: A review. *Chemical Engineering and Processing - Process Intensification* .2020;157: 10.1016/j.cep.2020.108148 10.1016/j.cep.2020.108148

[2] : Bioalcohol Reforming: An Overview of the Recent Advances for the Enhancement of Catalyst Stability. *Catalysts* .2020;10(6) : 10.3390/catal10060665 10.3390/catal10060665

# Response

**Published:** 2021-09-22

**Authors:** Paranjeet Lakhtaria

**Affiliations:** 24337, Portugal

The authors acknowledge these comments. The discussion section has been improved as recommended.

1. As suggested, a schematic of an APR reactor has been added and discussed in section ´Reactor types´. A diagram of an integrated APR with HT-PEMFC could not have been added; to the best of our knowledge, it has not yet been reported in literature.
2. CO-tolerance for both LT- PEMFCs and HT-PEMFCs have been added, based on numeric simulations, in the paragraph ´feasibility of reforming for fuel cell applications´.
3. The authors acknowledge this suggestion. Though briefly addressed in section Alkane formation, a dedicated table has been added concerning the by-products of the methanol APR.
4. The temperature range for steam reforming of alcohols has been corrected with the addition of suggested references.
5. Figure 4 has been improved as recommended.
6. A list of acronyms and nomenclature has been added at the beginning of the article.
